# Nanomaterial Functionalized Carbon Fiber-Reinforced Composites with Energy Storage Capabilities

**DOI:** 10.3390/nano15171325

**Published:** 2025-08-28

**Authors:** Venkatesh Gangipamula, Karamat Subhani, Peter J. Mahon, Nisa Salim

**Affiliations:** 1 School of Engineering, Swinburne University of Technology, Melbourne, VIC 3122, Australia; vgangipamula@swin.edu.au (V.G.); ksubhani@swin.edu.au (K.S.); 2 Aerostructures Innovation Research (AIR) Hub, Swinburne University of Technology, Melbourne, VIC 3122, Australia; 3 School of Science, Computing and Emerging Technologies, Swinburne University of Technology, Melbourne, VIC 3122, Australia; pmahon@swin.edu.au

**Keywords:** active surface area, carbon-based nanomaterials, carbon fiber, energy storage device (ESD), functionalization, structural supercapacitor (SSC)

## Abstract

We have demonstrated the fabrication of laminate composites with functional features to demonstrate energy storage capabilities. The present study investigates the surface modification of carbon fibers by coating dual materials of reduced graphene oxide (rGO) and cellulose-based activated carbon to enhance their energy storage capacitance for the development of structural supercapacitors. The dual coating on carbon fibers enabled a near 210-fold improvement in surface area, surpassing that of pristine carbon fibers. This formed a highly porous graphene network with activated carbon, resulting in a well-connected fiber–graphene-activated carbon network on carbon fibers. The electrochemical supercapacitor, fabricated from surface-functionalized carbon fibers, provides the best performance, with a specific capacitance of 172 F g^−1^ in an aqueous electrolyte. Furthermore, the symmetrical structural supercapacitor (SSSC) device delivered a specific capacitance of 227 mF g^−1^ across a wide potential window of 6 V. The electrochemical stability of the SSSC device was validated by a high capacitance retention of 97.3% over 10,000 cycles. Additionally, the study showcased the practical application of this technology by successfully illuminating an LED using the proof-of-concept SSSC device with G-aC/CF electrodes. Overall, the findings of this study highlight the potential of carbon fiber composites as a promising hybrid material, offering both structural integrity and a functional performance suitable for aerospace and automobile applications.

## 1. Introduction

The escalating concerns about the increasing carbon emissions in the transport sector have sparked considerable interest in innovative green energy storage solutions for future electric vehicles [1,2]. Among these energy storage solutions, supercapacitors (SC) possess a unique advantage, characterized by their higher power density, exceptional charge–discharge rates, and superior cycling performance, surpassing those of conventional batteries. Additionally, they offer greater energy density compared to traditional capacitors [3,4,5,6]. In recent years, research on energy storage composites (ESC) has gained attention for their ability to exhibit both structural load-bearing and energy storage capabilities [7]. This feature not only facilitates mass reduction, but also enhances payload capacity and range, making them suitable for future electric vehicles in both the automotive and aviation sectors, and its application extends to wearable and mobile electronics [8,9]. Among ESC variants [8,10,11], structural supercapacitors (SSC) with high surface area carbon-based electrodes are a suitable choice to fulfill the various energy storage applications due to their ability to exhibit an equitable amount of energy density, power density, and superior cycling performance [12,13,14]. However, the performance of SSC is a challenging factor that depends on the choice of electrode materials with higher surface area and a rich porous structure exhibiting electric double layer capacitor (EDLC) behavior to accumulate the charge at the electrode–electrolyte interface [6,15] and structural fibrous scaffolds, which can bear mechanical loads [16].

In SSC devices, a commonly used electrode material is carbon fiber (CF), due to its good electrical conductivity, along with a high strength-to-weight ratio, thermal stability, and chemical inertness [6,17]. Carbon fibers with a smaller diameter exhibit a higher specific surface area (SSA) and an improved surface reaction area, which enhances bonding with sizing materials [18]. For instance, carbon fibers averaging 10 μm in diameter demonstrate limited electroactivity and mass transport capabilities. Zeng et al. demonstrated in their study that the SSA increases from 11.75 m^2^ g^−1^ to 83.26 m^2^ g^−1^ as the diameter of electro-spun carbon fibers (ECFs) decreases from 1.819 μm to 0.282 μm. Moreover, the area resistance of ECFs rises from 8.61 mΩ cm^2^ to 15.72 mΩ cm^2^ with an increase in diameter. This is due to a reduction in areal density, which hinders inter-network electron transfer and results in a decline in capacitance from 8.00 × 10^−3^ F to 1.50 × 10^−3^ F as the diameter increases from 0.282 μm to 1.819 μm [19]. However, CFs’ diameter is not only the factor that influences the SSA, but it also depends on the existing impurities, morphology, and structure of CFs [20]. Nevertheless, the low active surface area (~0.2 m^2^ g^−1^) limits the capacitance of CFs, in turn yielding an inadequate performance of SSCs [21], which can be enhanced by the surface activation and functionalization of CFs with various materials with a high active surface area [21,22,23,24,25,26,27,28,29]. However, surface activation enhancement by physical processes mainly degrades the CF’s strength, and chemical methods involving reactive chemicals with high surface burn-off rates have the downside of affecting the mechanical properties of CFs [11,30]. For instance, the work by H. Qian et al. observed that the tensile strength of CFs treated with CO_2_, nitric acid, and concentrated KOH was reduced from 3300 ± 200 MPa to 2400 ± 250 MPa, 3100 ± 260 MPa, and 2600 ± 320 MPa, respectively [30]. Various investigations indicate that carbon-based materials, redox-active materials, and metal oxides can be grafted onto the surface of CFs, covering the inter and intra-tow spaces, contributing towards the improvement of surface area and mechanical reinforcement, simultaneously [4,15,31,32].

Likewise, graphene and its derivatives are extensively used as electrode material for supercapacitors due to their unique properties, such as a theoretical specific surface area of 2630 m^2^ g^−1^, specific capacitance of 550 F g^−1^, and excellent Young’s modulus of 1.1 TPa [33,34,35]. Of the various coating techniques, hydrothermal processing is a binder-free and more facile one-step method to functionalize CFs without largely affecting mechanical strength [36]. Previously, our group developed an SSC device with the self-assembly of a graphene aerogel (GA) onto CF electrodes, yielding a capacitance of 92.5 F g^−1^ at 1 mV s^−1^, thereby increasing the electrochemical properties of carbon fiber-reinforced polymer (CFRP) composite by 500 times more than the neat composite. The SSC device exhibited a capacitance of 56 mF g^−1^, without any activation of the CFs [28]. A recent study by Zhou et al. confirmed that the specific capacitance of the SSC can be further enhanced by CF fabric activation [3]. In their work, Yuan et al. have fabricated free-standing electrodes for an asymmetric supercapacitor application with 3D graphene aerogel (GAG) from reduced graphene oxide (rGO), coupled with carbon nanotubes (CNT) as spacers and polyacrylonitrile (PAN) nanofibers as the supporting structure. To improve pseudocapacitance, they coated the films with polypyrrole (PPy), resulting in a positive electrode made of RGO/CNT/PAN-PPy and with nitrogen-doped rGO/CNT/carbon nanofibers (NRCC) as the negative electrode after undergoing pyrolysis. The as-prepared supercapacitor exhibited a specific capacitance of 716 F g^−1^, with an energy density of 60.6 Wh Kg^−1^ at 850.2 W kg^−1^ [37].

When examining the nominal output of electrochemical performance, it can be attributed to two key factors. Firstly, there is an insufficient quantity of active material integration on the CF’s surface, and secondly, there is the presence of unstable or inadequately bonded active material on the CFs’ surface. This latter issue can lead to a material loss on the modified electrodes when electrolytes are encountered during the fabrication of SSCs [21]. Moreover, surface activation or single material coating is insufficient to enhance the electrochemical performance, so doping the active material with a secondary conductive material will contribute much more enhancement to the surface area. For example, Javaid et al. have developed a high-performing SSC with modification of a CF fabric by impregnating it with a precursor solution of carbon aerogel (CAG) suspended with 5% of graphene nanoplatelets (GNP), acting as nano reinforcements with an improved surface area of 223 m^2^ g^−1^. This produced an SSC with a specific capacitance of 400 mF g^−1^, but the performance was limited by the conductivity difference between electrolytes and electrodes [27].

Another promising carbonaceous material is activated carbon (aC), which can be derived from naturally available materials at low cost and exhibits a hierarchical porous structure with excellent conductivity and specific capacitance [21,38,39]. Therefore, using biomass as a precursor to produce porous carbons for electrode materials in SCs is common because of their ability to self-dope with heteroatoms (boron, nitrogen, oxygen, sulfur, etc.), which results in a larger SSA and forms active sites that contribute towards pseudocapacitance [40,41]. Enhancement of the surface area, along with porosity, is a necessary aspect that will have a synergistic effect on the improvement of the electrochemical performance of porous carbon, due to increased charge accumulation at the electrolyte–electrode interface, and higher pore volume, which facilitates faster ionic movement [42]. Among various strategies, chemical activation using alkaline reagents (like KOH, NaOH, H_3_PO_4_, ZnCl_2_, etc.) is widely used to generate porous carbons with increased pore volume of various sizes [43]. However, higher mesoporous volume with pore sizes matching that of ions facilitates faster ion transport kinetics compared to micropores, which restrict ionic movement while providing higher SSA [41]. The porous structure of biomass precursors can be enhanced and controlled by coupling chemical activation with KOH and regulated high-temperature carbonization.

For example, Wang et al. found that treating wheat husk biomass with chloride salts during pre-carbonization increased carbon content and reduced oxygen release during KOH activation at 700 °C, which introduces an adequate amount of mesopores into the porous carbon network, along with improving SSA. Their porous carbon electrodes achieved a gravimetric capacitance of 402 F g^−1^ at 1.0 A g^−1^. A symmetrical supercapacitor made with these electrodes demonstrated a specific capacitance of 346 F g^−1^ at 1.0 A g^−1^ and retained 98.59% of its initial capacitance after 30,000 cycles at 5.0 A g^−1^ [42]. In a recent study by Rajivgandhi et al., activated carbon from foxtail millet (FMCA) with superior SSA and porous structure was synthesized via KOH activation at a pre-carbonization temperature of 500 °C, followed by carbonization at a higher temperature of 800 °C under inert atmospheres. The FMCA//FMCA symmetric device achieved a capacity of 82.94 F g^−1^ at 0.5 A g^−1^ and maintained a remarkable 94.89% capacity retention after 5000 cycles [43]. In another study by Nazhipkyzy et al., the process of hydrothermal pretreatment on sawdust precursors (karagash and pine) was carried out, followed by KOH activation at high temperatures to produce an activated carbon. The results were promising, as the activated carbon derived from karagash and pine sawdust exhibited the highest capacitance of 202 F g^−1^ and 161 F g^−1^ at a scan rate of 5 mV s^−1^. Additionally, these materials demonstrated significant energy densities, measuring 26.0 Wh kg^−1^ and 22.1 Wh kg^−1^, respectively [44]. Furthermore, the presence of aC particles in the graphene network has the advantage of restraining the restacking among graphene sheets due to π-π interactions, which negatively affects electrochemical performance [45].

In the present work, a novel approach is demonstrated for directly assembling both reduced graphene oxide (rGO) sheets and cellulose-based aC onto CF woven fabric, thereby enhancing the conductivity and surface area of CF-based electrodes. To the best of our knowledge, for the first time, we have demonstrated a two-step modification process by combining the hydrothermal pretreatment to coat GO on CF fabric, forming rGO/CF, followed by application of pre-processed G-cellulose-based (G-C) slurry with KOH as chemical activator, and subjecting the G-C-KOH slurry-coated rGO/CF to regulated high-temperature carbonization in inert atmosphere to form a porous rGO-aC composite structure on CFs’ surface. Our study confirmed that the synergistic effect of rGO and cellulose-based aC coating on CFs enhances the surface area and porosity, thereby improving the electrochemical performance of the as-prepared SSC device. This study further confirms that the porous network of rGO-aC (simply G-aC) extends into the solid polymer electrolyte (SPE) at the electrode–electrolyte interface. Furthermore, the G-aC/CF electrodes provide feasibility for better ionic movement through the porous and interconnected carbon network. Finally, a proof-of-concept symmetrical SSC device was fabricated using G-aC/CF electrodes sandwiching two layers of cellulose paper as the separator, and impregnated with an SPE to power an LED for 15 min.

## 2. Experimental Work

### 2.1. Materials

Electrodes were constructed from plain weave Dowaksa A-38 3k carbon fabric (Play with carbon, CF, Bulltech Pty Ltd., NSW, AU) after surface modifications, graphene oxide (1%, GO, supraG International Pty Ltd., VIC, AU), and graphene paste (20 *w*/*w*, First Graphene Ltd., WA, AU), with a separator being cellulose paper (wood fiber—Kimtech science, Kimberly-Clark Professional, NSW, AU). The chemicals acetone, nitric acid (98%), ethylene glycol (EG) (99.9%), cellulose (Nano form), potassium hydroxide (90%—flakes), hydrochloric acid (32%, HCl), polyethylene glycol diglycidyl ether (PEGDGE), triethylenetetramine (TETA), 1-ethyl-3-methylimidazolium bis(trifluoromethylsulfonyl)imide (EMIMTFSI), and lithium bis(fluorosulfonyl)imide (LiFSI) were supplied from Sigma-Aldrich, Merck Life Science Pty Ltd., VIC, AU. All chemicals were used as received.

### 2.2. Graphene Oxide Coating on Carbon Fiber Fabrics (rGO/CF)

**Figure 1:**
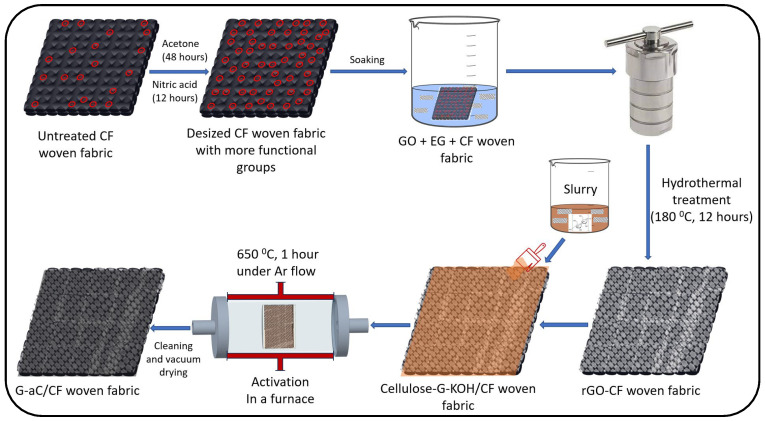
Schematic of process steps to prepare G-aC/CF fabrics.

Desized carbon fiber fabrics were cut into individual pieces and dried in the oven for 30 min at 60 °C. Diluted graphene oxide (GO) sheets in water were sonicated for 1 h to obtain a homogeneous solution with a 3 mg mL^−1^ concentration. This was followed by magnetic stirring for 30 min after adding 10 mL of ethylene glycol for every 60 mL of the GO solution. Each fabric was soaked in the GO + EG solution for 24 h, with a total of 6 fabrics being prepared. Individual fabrics were transferred into an autoclave vessel along with the solution, and the hydrothermal method was performed at 180 °C for 12 h, as shown in Figure 1. After completing the heating cycle, the vessel was allowed to cool to room temperature. The residual water was carefully disposed of, and the coated fabrics were transferred into separate beakers covered with parafilm to avoid airborne contaminants. The beakers were then placed in a freezer for 48 h. After removal from the freezer, the beakers were placed in a desiccator for vacuum drying for 24 h. These rGO-coated fabrics (rGO/CF) had nearly 3 wt.% of rGO bonded to the surface of the CFs and were transferred into plastic trays and enclosed to avoid moisture absorption.

### 2.3. Graphene, Cellulose, and KOH Slurry Preparation

**Figure 2:**
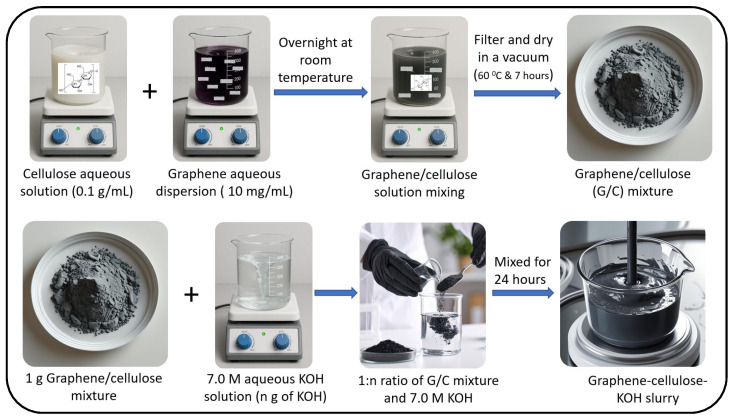
Preparation of graphene–cellulose–KOH slurry.

A graphene–cellulose–KOH slurry was synthesized using the process shown in Figure 2. To obtain a certain volume of graphene dispersion solution with a concentration of 10 mg mL^−1^, 20 mL of water was added for every 1 g of 20% *w*/*w* graphene paste and sonicated for 1 h to disperse the graphene sheets. Simultaneously, 1 g of the nano form of cellulose powder was mixed with 10 mL of water and subjected to magnetic stirring for 1 h. Both the graphene dispersion solution and cellulose solution were mixed overnight at room temperature. Subsequently, the combined solution was vacuum filtered and dried in a vacuum oven at 60 °C for 7 h. The resulting mixture of graphene and cellulose consisted of granules that were subsequently crushed. This mixture was then processed using a mesh sieve to obtain a fine powder. Here, two compositions of graphene dispersion solution and cellulose solution were considered to obtain the two concentrations of powder, that is, one with a 1.5:1 ratio and another with a 1:1 ratio of graphene dispersion solution and cellulose solution mixtures, yielding P_1_ and P_2_ graphene/cellulose mixture powders, respectively. A 7 M KOH solution was prepared, and for every 1 g of the P_1_ powder mixture, the required amount of 7 M KOH solution (n) was assessed to be 4 g, and for P_2_, it was 6.5 g to obtain a slurry form. Then, magnetic stirring was performed for 24 h to obtain homogeneous graphene–cellulose–KOH slurries S_1_ and S_2_, respectively.

### 2.4. Preparation of CF Fabric Electrodes with Graphene and Activated Carbon

The graphene–cellulose–KOH slurry was brush-coated onto the dry rGO/CF fabrics to ensure complete coverage. The coated fabrics were then allowed to dry at room temperature for 72 h. After drying, the graphene–cellulose–KOH-coated rGO/CF fabrics were placed in a furnace, where they were heated at a controlled rate until reaching 650 °C for 1 h under an argon atmosphere with a flow rate of 5 L min^−1^ (the furnace was programmed to increase the temperature by 10 °C every minute, taking a total of 65 min to reach 650 °C, followed by 1 h of treatment at that temperature). High-temperature carbonization causes the formation of aC; excessive G-aC was collected and used to perform BET surface characterization. The samples were allowed to cool to room temperature, and the pH of the samples was adjusted to pH 7.0 by washing with 0.1 M HCl solution and deionized water. The obtained coated carbon fiber fabrics with graphene and activated carbon (G-aC/CF) were dried in a vacuum oven at 130 °C for 20 h. After the cleaning and drying steps, the firmly bonded coating material accounts for nearly 14 wt.% of loading on CFs in the case of S_2_. The complete process involved in preparing G-aC/CF fabrics is illustrated in Figure 1.

### 2.5. Fabrication of Symmetrical Structural Supercapacitor (SSSC)

**Figure 3:**
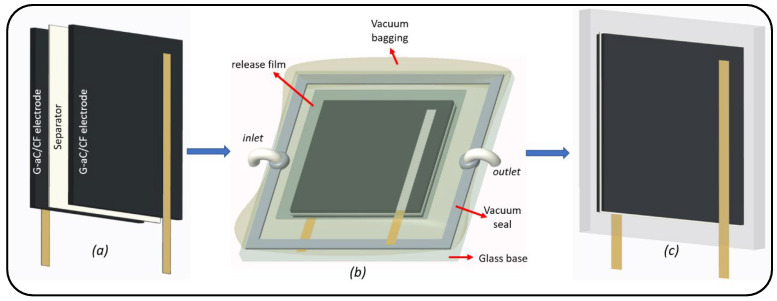
Process of SSSC device fabrication; (**a**) G-aC/CF electrodes (6 × 6 cm^2^) and separator, (**b**) VRTM with vacuum bagging and SPE infusion, (**c**) demolded SSSC device with G-aC/CF electrodes.

**Figure 4:**
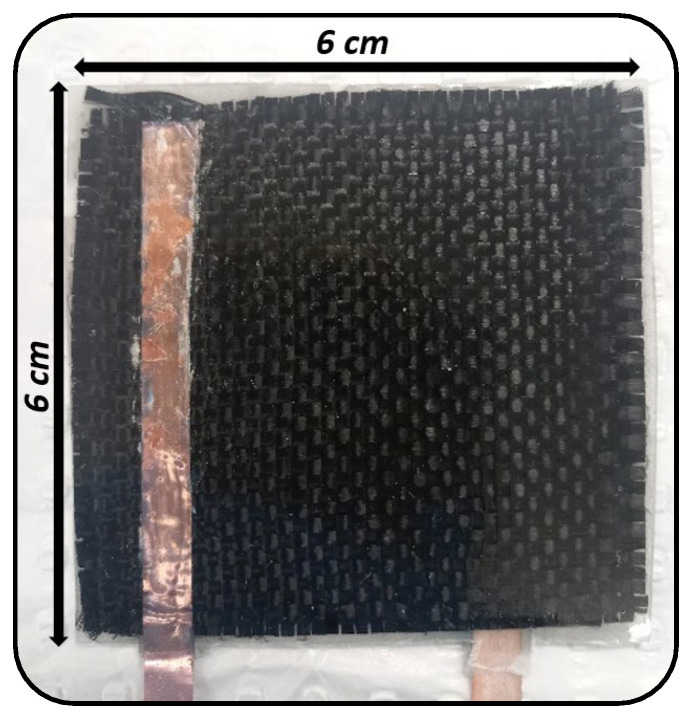
Depiction of a fabricated SSC device.

A symmetrical SSC was fabricated using two (6 × 6 cm^2^) fabrics of G-aC/CF electrodes separated by two layers of cellulose paper and impregnated with solid polymer electrolyte (SPE) using a vacuum-assisted resin transfer molding (VRTM) setup, as shown in Figure 3. Before performing VRTM, the two electrode fabrics were attached with conductive copper tape as current collectors by applying silver ink at the interface of the fabric and conductive tape. The SPE was formulated by combining 83 wt.% of the polymer (PEGDGE) with 10 wt.% of the ionic liquid (1-Ethyl-3-methylimidazolium bis(trifluoromethylsulfonyl)imide) and 2 g of an ionic salt (lithium bis(fluorosulfonyl)imide), which were mixed using a magnetic stirrer for one hour. Subsequently, 7 wt.% of the crosslinker TETA was added, and the mixture was stirred for an additional 7 min. Demolding of the fabricated SSSC from vacuum bagging was carried out after allowing it to be cured at room temperature for 12 h, after which it was further cured in an oven at 80 °C for 24 h to obtain the SSSC, as depicted in Figure 4. This SSSC was evaluated for its electrochemical performance.

### 2.6. Material Characterizations

A Scanning Electron Microscope (SEM; ZEISS SUPRA 40 VP) was utilized at an operating voltage of 5 kV to characterize the microstructures of desized CF, rGO/CF, and G-aC/CF fiber sample materials. Raman spectroscopic measurements were performed using a WiTec Alpha 300R confocal Raman microscope, spanning a wavenumber range from 0 to 4000 cm^−1^, with a 514 nm wavelength laser source. The laser operated at a power of 50 mW for an exposure duration of 20 s. The chemical compositions of the desized CF, rGO/CF, and G-aC/CF fiber samples were analyzed using X-ray photoelectron spectroscopy (XPS) with a Kratos AXIS NOVA spectrometer (Kratos Analytical, Inc., Manchester, UK) equipped with a monochromatic Al Kα X-ray source. During the analysis, the typical pressure was maintained at 2.3 × 10^−8^ mbar. Deconvolution of the spectra was performed using CASA XPS software (Version 2.3.19PR1.0). X-ray diffraction (XRD) analysis was conducted using a Bruker X-ray Solutions D8 ADVANCE ECO system, functioning at 40 kV and 40 mA with Cu-Ka radiation (λ = 0.154 nm) in a wide-angle range of 5 to 60°. The surface area of the desized CF, rGO/CF, and G-aC/CF fiber samples was evaluated through the Brunauer–Emmett–Teller (BET) and Barret–Joyner–Halenda (BJH) methods, employing a TriStar II Plus Series Micrometrics instrument.

### 2.7. Electrochemical Measurements

**Figure 5:**
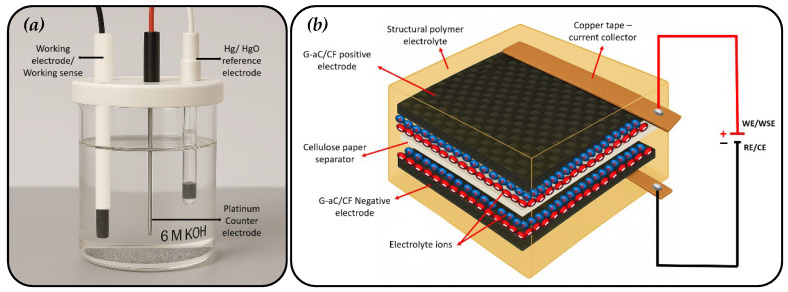
Depiction of cyclic voltammetry tests; (**a**) modified CF electrodes in a 3-electrode setup, (**b**) two-electrode testing of the fabricated SSSC device with G-aC/CF electrodes.

Cyclic voltammetry (CV) analysis, galvanostatic charge/discharge (GCD), and electrochemical impedance spectroscopy (EIS) measurements for all modified CF samples and the SSSC device were conducted using a Gamry Instruments Interface 5000e (USA). Single-electrode analysis was carried out in a three-electrode cell, utilizing a platinum counter electrode and an Hg/HgO reference electrode immersed in a 6 M KOH aqueous solution, as illustrated in Figure 5a below. The desized CF, rGO/CF, and G-aC/CF fiber samples served as the working electrodes during their respective tests. The tests were conducted over a potential range of 1.5 V, with various scan rates of 1, 5, 10, 20, 50, and 100 mV s^−1^, and GCD analysis was executed at different current densities ranging from 0.5, 0.75, 1.25, and 2.5 mA g^−1^. A two-electrode cell system was employed to test the SSSC device operating with a potential range of 6 V. Once potential is applied across the SSSC device, charge separation will occur at the interface of SPE and G-aC/CF electrodes, as shown in Figure 5b. The CV experiment for the SSSC device was conducted at various scan rates, specifically 1, 5, 10, 20, 50, and 100 mV s^−1^, and GCD analysis was performed at current densities of 0.5, 0.75, 1.25, and 2.5 mA g^−1^. The EIS analysis was carried out in the frequency range of 100 kHz–0.1 Hz with an AC voltage amplitude of 10 mV for both modified CF electrodes and the SSSC device. The calculations of the gravimetric capacitance (F g^−1^), energy density (Wh kg^−1^), and power density (kW kg^−1^) were carried out using the appropriate formulas [11,46], as follows.

(1) $$C_{CV}=\frac{Q}{2\;∆V\;m\;\nu }$$

(2) $$C_{GCD}=\frac{I\;∆t}{m\;∆V}$$

(3) $$E=\frac{1}{2}C\times V^{2}$$

(4) $$P=\frac{E}{∆t}\times 3600$$

Here, *C_CV_* and *C_GCD_* represent the gravimetric capacitance (F g^−1^) of the cyclic voltammogram and charge–discharge curves, respectively, *Q* refers to the cumulative charge obtained from the cyclic voltammogram, Δ*V* indicates the potential window (V), *ν* denotes the scan rate (mV s^−1^), *m* is the total mass of the electrode (g), *I* is the charge–discharge current, *E* stands for energy density, *P* represents power density, and Δ*t* signifies the cell discharge time.

## 3. Results and Discussion

**Figure 6:**
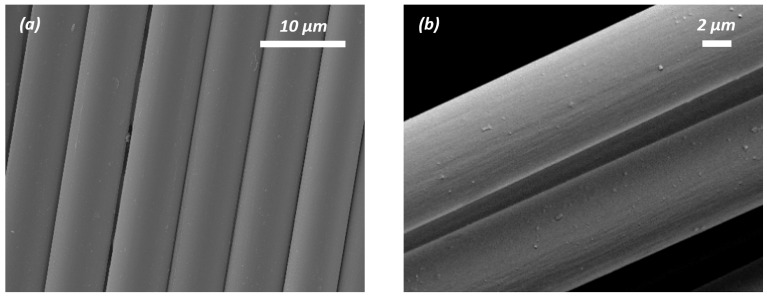

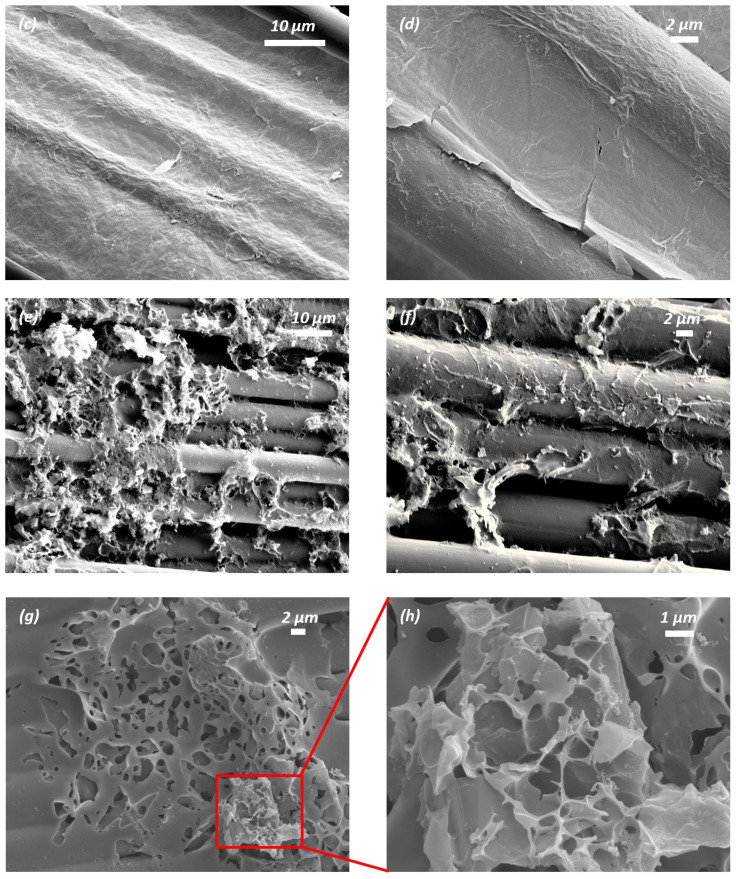
SEM imaging of (**a**) pristine CF, (**b**) desized CF, (**c**,**d**) rGO/CF, (**e**,**f**) G-aC/CF_1_, (**g**,**h**) G-aC/CF_2_.

The morphologies of pristine CF, desized CF, grafted rGO/CF, and graphene-activated carbon (G-aC)-coated CF were characterized by SEM, as shown in Figure 6a–h. Along with these, SEM imaging (see Figure S1) of nano-form cellulose is presented in the Supporting Information. While performing the desizing procedure, the CF surface (see Figure 6a) is etched by the acetone and nitric acid treatment for 48 and 12 h, respectively, to form a coarse surface with visible grooves depicted in Figure 6b. These grooves can enable an enhancement in oxygenated groups to facilitate the bonding with reduced GO coatings onto desized CFs [47].

Figure 6c,d shows the formation of rGO nanosheets covering the surface of the CFs. The hydrothermal process induces the wrapped and well-connected nanosheets of the rGO network bonded to the surface of the CF due to van der Waals forces via π-π interactions [47]. The second coating was applied using the slurry S_1_, followed by high-temperature furnace activation, which yielded a poor form of coated sample G-aC/CF_1_, as shown in Figure 6e,f, on which agglomerated graphene sheets and aC clusters are formed with poor porous network reinforcement of the inter and intra-tow spaces; hence, characterization of this sample is limited to SEM and cyclic voltammetry (CV) observations. A second coating was simultaneously performed using prepared slurry S_2_, followed by high-temperature furnace activation of another substrate of rGO/CF that led to the formation of the highly porous network of graphene and activated carbon covering the surface of the CFs and reinforcing the inter-tow and intra-tow spaces of the CF fabric. The resulting well-connected fiber–graphene-activated carbon network of sample G-aC/CF_2_ is presented in Figure 6g,h. It can also be observed from Figure 6g that there are a few stacked layers of graphene (as marked) on the CFs due to thermal reduction. As G-aC/CF_2_ offers an improved surface, it will be denoted as G-aC/CF in subsequent characterization test results and discussions, excluding CV, GCD, and EIS analysis of fiber electrodes.

**Figure 7:**
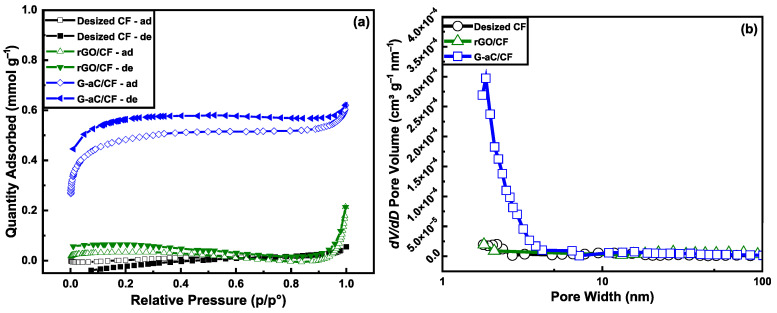
(**a**) Nitrogen adsorption (ad) and desorption (de) isotherms and (**b**) Pore size distribution of desized CF, rGO/CF, and G-aC/CF.

The surface area, pore size, and volume are the prime driving factors for better electrochemical performance of electrodes in a supercapacitor [48]. Surface analysis using BET was performed on desized CFs, rGO/CF, and G-aC-coated CFs to observe N_2_-sorption, which revealed the surface areas (see Figure 7a) and pore size distribution (see Figure 7b). The results demonstrate that the desized CF exhibits a surface area of 0.16 m^2^ g^−1^, which is enhanced to 2.36 m^2^ g^−1^ due to the accumulation of rGO sheets on the CF surface in the case of rGO/CF. Furthermore, the surface analysis of G-aC powder exhibits a surface area of 64.27 m^2^ g^−1^ and a porous structure with an average pore size of 4.15 ± 0.25 nm, as provided in Table 1 (N_2_-sorption isotherms and pore size distribution graphs are presented in Figure S2). When the G-aC deposition formed on the CFs, there is an increase in the surface area to 34.21 m^2^ g^−1^, which is more than 210 times greater than the uncoated CFs, with an average pore size of 2.25 nm. 

However, this enhancement in the surface area is minimal, and it is important to recognize that this significant alteration in surface parameters can greatly influence electrochemical, as demonstrated in a study where the modification was performed on CF with a varied coating density of PANI [32]. Moreover, the reason for the contraction of the G-aC network is due to the incorporation of CFs, forming a high density and surface area for the G-aC/CF electrodes [24]. This can be correlated with the change in surface morphology of G-aC-modified CFs observed in SEM imaging, possessing porous and interconnected structures that also contribute towards the enhanced capacitance of the coated electrodes. Table 1 indicates that the pore volume increases after each coating step, with a 28.6% increment observed between the desized CFs and rGO/CF with a loading of 3.2 wt.% of rGO, rising from 0.0015 cm^3^ g^−1^ to 0.002 cm^3^ g^−1^. Additionally, after synthesizing the second coating on the rGO/CF substrate, forming G-aC/CF with active material loading of 13.8 wt.%, there is a remarkable enhancement in pore volume by 161.9%, increasing from 0.002 cm^3^ g^−1^ to 0.019 cm^3^ g^−1^. Moreover, the amount of mesopores in G-aC/CF increased significantly, with an average size of 2.25 nm (see Figure 7b), which is vital for the enhancement of the ionic movement contributing towards electrochemical properties [49]. KOH chemical activation coupled with controlled high-temperature furnace treatment in our coating process provided the feasibility to enhance the volume of mesopores nearly 13 times on the surface of G-aC/CF electrodes than desized CF, making the SSA more accessible to ions and contributing towards the faster ionic transportation [42,50]. 

**Figure 8:**
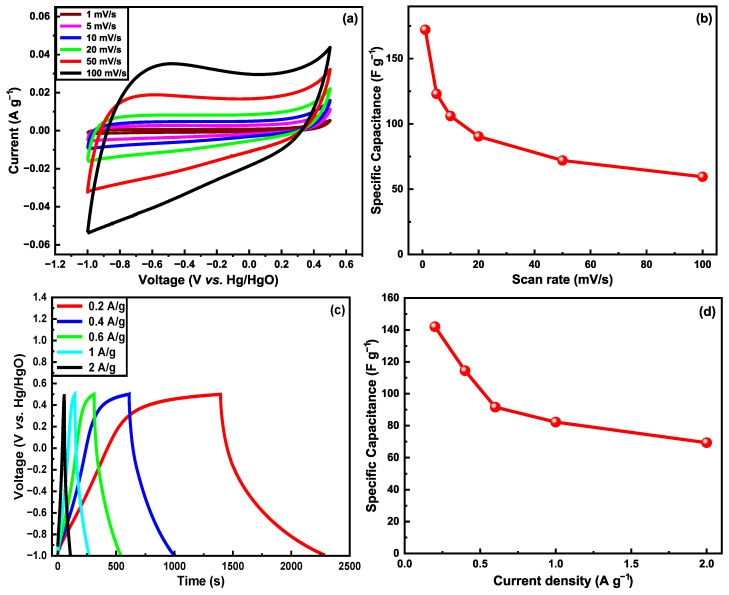

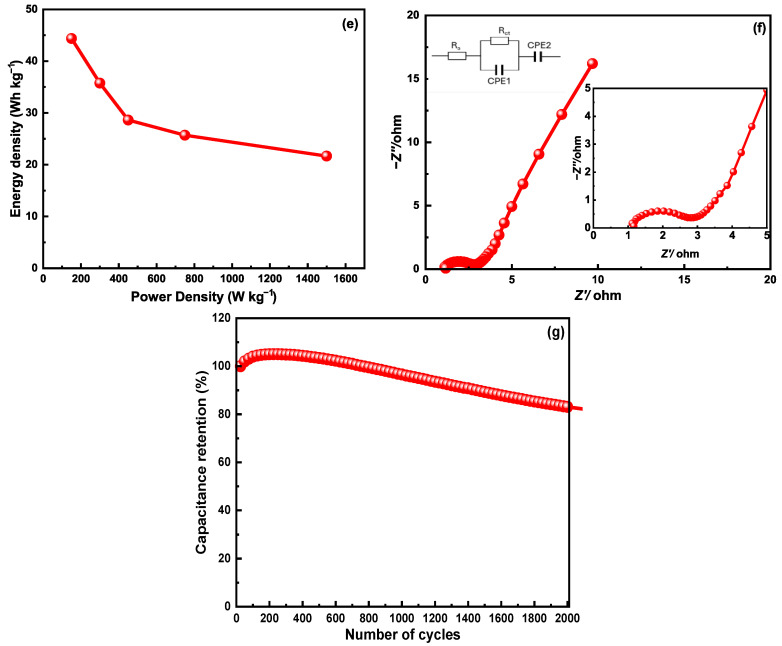
(**a**) Cyclic voltammograms of G-aC/CF fiber electrode at different scan rates, (**b**) specific gravimetric capacitance at different scan rates, (**c**) GCD curve of G-aC/CF at various current densities, (**d**) specific gravimetric capacitance of G-aC/CF at different current densities, (**e**) Ragone plot for G-aC/CF fiber electrode, (**f**) electrochemical impedance spectroscopy measurement of G-aC/CF fiber electrode in 6 M KOH electrolyte, (**g**) cycling performance of G-aC/CF fiber electrode.

The electrochemical performance (Figure 8) was evaluated with a three-electrode system using a platinum counter electrode and an Hg/HgO reference electrode in a 6M KOH electrolyte solution. The coated fibers were used as the working electrode. Cyclic voltammetry studies were conducted on various coated fiber electrodes, including G-aC/CF_1_, G-aC/CF_2_, and rGO/CF fiber tows, weighing 10 mg, 4.3 mg, and 4 mg (inclusive of both active material and CFs), respectively, along with desized carbon fibers (CF), weighing 15 mg. The analysis was conducted across a voltage range from −1 to +0.5 V, with scan rates varying between 1 and 100 mV s^−1^. Among the materials tested, desized CF demonstrated a gravimetric capacitance of 9.38 F g^−1^ at a scan rate of 1 mV s^−1^. In comparison, the rGO/CF fibers exhibited an even greater capacitance of 22.54 F g^−1^ under the same conditions. Remarkably, the G-aC/CF_1_ fibers, despite their suboptimal material coating, achieved a gravimetric capacitance of 29.93 F g^−1^ at a scan rate of 1 mV s^−1^, which is almost three times that of desized CFs (refer to Figure S3a–c).

Furthermore, analysis of the CV data for sample G-aC/CF_2_, at lower scan rates (see Figure 8a), shows a nearly rectangular shape, denoting an electric double layer capacitor (EDLC) response; as the scan rate increases from 1 to 100 mV s^−1^, the CV curve changes to the quasi-rectangular shape representing the existence of both EDLC and pseudocapacitive charge storage features [51]. Compared to other samples, the highest gravimetric capacitance of 172 F g^−1^ was observed for the G-aC/CF_2_ fiber electrode at a scan rate of 1 mV s^−1^. Here, various reasons have contributed towards the enhanced electrochemical performance of G-aC/CF_2_ fiber electrodes after performing the second stage of the coating procedure. For instance, the porous carbon network on the CFs’ surface was formed due to the surface etching of graphene and aC, creating pores on their surfaces by KOH activation at elevated temperatures [49,52,53], leading to a 210-fold increment in SSA and a 161.9% improvement in pore volume. This porous carbon network consists of a very high amount of micropores and mesopores, as shown in Figure 7b. Micropores and mesopores provide extra ionic sites for electrolyte ions, enhancing capacitance. Furthermore, it provides additional ionic transport channels to have electrolyte ions reach the inner sites more efficiently [54,55]. Additionally, improved surface area provides a better electrolyte/electrode interface, which is critical for high charge transfer [56]. Furthermore, the mitigation of restacking of graphene sheets by introducing aC as spacers has contributed towards improved SSA [53]. Moreover, according to the insights reported by Yin et al., even after high-temperature treatment during the second-stage coating process in this work, there are still oxygen-containing functional groups that exist on the surface of graphene, as confirmed through XPS observations. These functional groups can exhibit the capacity to undergo electron transfer during the charging and discharging cycles, thereby facilitating the phenomenon of pseudocapacitance, contributing towards the improved capacitive nature of the electrodes [57]. 

As is common, the capacitance values reduced as the scan rate increased, as illustrated in Figure 8b. The phenomenon in porous carbon-based materials is related to micropore blockage, ion diffusion reduction, and ionic resistance effects that are induced at higher scan rates due to the increase in current, while at low scan rates, ions will have enough time to intercalate/deintercalate with the electrode’s active surface area [28,58]. However, it is worth noting that during the second coating process, the varying amounts of graphene in the synthesized slurry significantly impacted the SSA, porous structure, and electrochemical performance. In S_1_, a higher concentration of graphene led to the formation of clusters due to the restacking of graphene sheets, which adversely affected both the SSA and porous structure, as evidenced by the SEM images (Figure 6e,f). Conversely, by reducing the graphene content in S_2_, a uniform coating was observed on the carbon fibers, with graphene sheets being effectively segregated by the spacer material aC. This adjustment contributed to the development of a three-dimensional porous structure (Figure 6g,h), thereby enhancing the pore volume and SSA, which ultimately improved capacitance.

Additionally, galvanostatic charge–discharge (GCD) and electrochemical impedance spectroscopy (EIS) analysis were performed on the coated fibers, with an exceptional performance (G-aC/CF_2_) observed during the CV studies. The GCD analysis was conducted at various current densities ranging from 0.2 A g^−1^ to 2 A g^−1^, across a voltage window of −1 to 0.5 V, as illustrated in Figure 8c,d. The G-aC/CF_2_ fiber demonstrated the highest gravimetric capacitance of 142 F g^−1^ at a current density of 0.2 A g^−1^ and a lower capacitance of 69.3 F g^−1^ at 2 A g^−1^, indicating a 50% decrease in capacitance with increasing current density. The GCD plot reveals an imperfect triangular shape, showcasing the favorable electrical double-layer capacitor characteristics [59]. This non-linear portion of the curve further confirms the presence of pseudocapacitance at a certain level in alignment with CV graphs [60]. At a current density of 0.2 A g^−1^, the energy density and power density achieved by the G-aC/CF_2_ fiber were 44.4 Wh kg^−1^ and 150 W kg^−1^ (see Figure 8e), respectively.

The EIS response was recorded for the G-aC/CF_2_ electrode in a three-electrode setup to measure the impedance of the electrode using equivalent circuit analysis. The Nyquist plot of the G-aC/CF_2_ electrode with an equivalent circuit diagram in the insert is presented in Figure 8f. It can be observed in the low frequency area that the portion of the curve is almost perpendicular to the Z’-axis, indicating that there is fast ionic diffusion at the interface of the G-aC/CF_2_ electrode and electrolyte, induced by the existence of higher porosity of the G-aC/CF_2_ electrode [61]. Moreover, the formation of a quasi-semicircle with a small diameter in the high frequency region (enlarged in the insert) signifies the lower charge transfer resistance between the electrode and electrolyte interface [40]. In the circuit diagram, R_s_, R_ct_, and constant phase elements CPE1 and CPE2 denote solution resistance, charge transfer resistance, double layer capacitance, and porosity of the G-aC/CF_2_ electrode [11]. The measurements reveal that R_s_ and R_ct_ of the G-aC/CF_2_ electrode are observed to be 1.01 Ω and 2.05 Ω, which are in good agreement with the literature [40,62]. A lower value of R_ct_ signifies the enhancement of the rate capability of the G-aC/CF_2_ electrode due to the existence of a porous structure on it [40].

**Figure 9:**
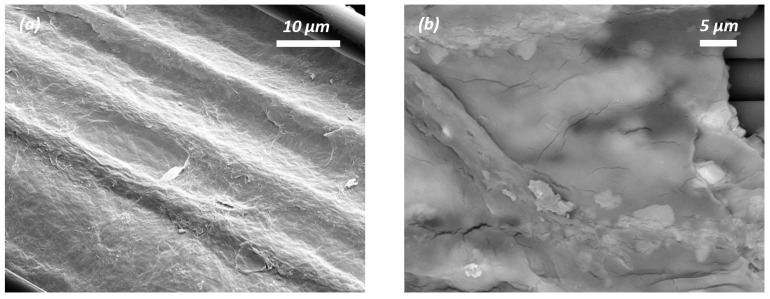

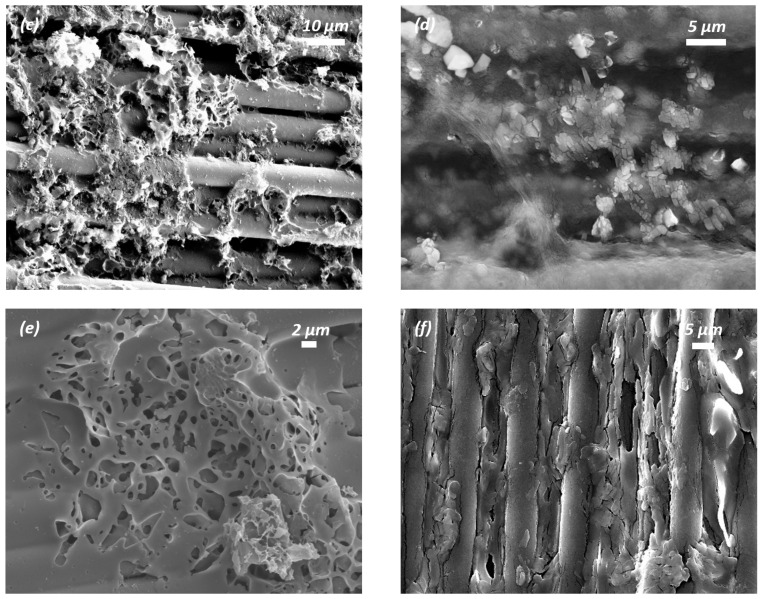
SEM imaging of samples before and after electrochemical analysis; (**a**,**b**) rGO/CF, (**c**,**d**) G-aC/CF_1_, (**e**,**f**) G-aC/CF_2_.

Likewise, cyclic voltametric studies demonstrated that the fiber electrode shows an outstanding capacity retention of 83.04% over 2000 cycles at 100 mV s^−1^, as shown in Figure 8g. Following the completion of electrochemical studies of coated fiber electrodes in a three-electrode setup with 6M KOH electrolyte, SEM images of the samples were obtained to illustrate the corrosive effect of KOH on the electrodes. Figure 9b demonstrates the formation of cracks on the rGO sheets for the rGO/CF sample, while Figure 9d,f of the G-aC/CF samples reveal that the micro-porous structure on the surface of the CF is disrupted by the etching of the coated layers, along with the development of cracks in the coating material. This clarifies that the observed reduction in capacity retention during cyclic studies of G-aC/CF_2_ fiber electrode can be attributed to the corrosive effects of KOH, which led to the etching of the coating material.

**Figure 10:**
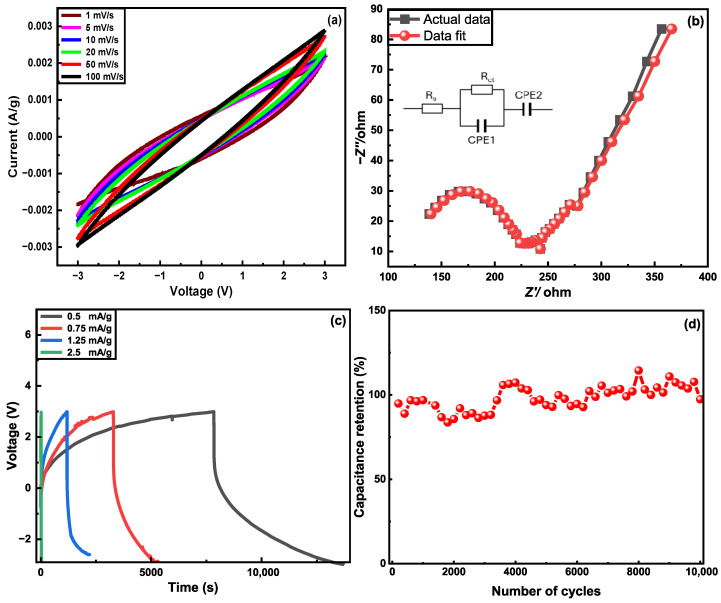
Electrochemical analysis of the SSSC with G-aC/CF electrodes; (**a**) cyclic voltammogram at different scan rates, (**b**) electrochemical impedance spectroscopy measurements, (**c**) GCD curve at various current densities, (**d**) cycling performance at 100 mV s^−1^.

The symmetrical structural supercapacitor, constructed with G-aC/CF electrodes, exhibited the highest capacitance of 155 mF g^−1^ at a scan rate of 1 mV s^−1^ over a potential range from −3 to 3 V, from CV observations, as depicted by Figure 10a. The EIS response was analyzed using equivalent circuit analysis for the SSSC device in a two-electrode configuration. From the Nyquist plot, along with the equivalent circuit diagram, as shown in Figure 10b, it can be noted that a segment of the curve is nearly perpendicular to the Z’-axis in the low-frequency region, indicating the EDLC behavior of the SSSC device (which is similar to the EIS response observed in [23] SSC devices). The larger diameter of the quasi-semicircle formed in the high-frequency region indicates that there is a high charge transfer resistance at the interface between the electrode and electrolyte due to the solid phase of SPE. The represented electrolyte resistance (R_s_) and charge transfer resistance (R_ct_) from the circuit diagram are measured to be 117.01 Ω and 108.67 Ω, respectively. These elevated values are attributed to the restricted movement of ions in the SPE, which is directly related to the diminished electrochemical performance of the SSSC device [23,24,63]. Further GCD analysis of the SSSC device is conducted at different current densities ranging from 0.5 to 2.5 mA g^−1^, as illustrated in Figure 10c. The device demonstrated its peak performance at a current density of 1.25 mA g^−1^, achieving an energy density of 0.99 Wh kg^−1^ and a power density of 3.5 W kg^−1^, with a capacitance value of 227 mF g^−1^ across a potential window of 6 V. This symmetrical SSC device has showcased an impressive operational potential window of 6 V, exceeding findings from other studies [64], such as one that reported a maximum operational potential of 2.5 V for an asymmetrical SSC constructed from modified carbon fiber cloths. The remarkable 6 V operational potential window achieved by our SSSC device highlights the significance of electrolyte and electrode material selection, as suggested by the existing literature. Electrode materials that incorporate heteroatom-doped carbon and exhibit a defective graphite structure have shown promising results in harnessing high potential windows [65]. For instance, Wang et al. achieved a potential window of 1.8 V using N-doped activated carbon sheets in a symmetric capacitor. This impressive performance can be attributed to the electrode’s unique porous structure and its heteroatom-doping characteristics [66]. The use of non-aqueous electrolytes contributes to broad potential windows, primarily because they contain lower concentrations of H^+^ and OH^−^ ions, which help to increase the overpotential for gas evolution, and consequently widen the working voltage of the device. Furthermore, the presence of the ionic liquid within the electrolyte enhances short-range coulombic interactions, affecting relative permittivity and specific conductance. This alteration leads to modifications in specific capacitance and electrolyte resistance, enabling a broader range of high capacitive potential windows [67].

**Figure 11:**
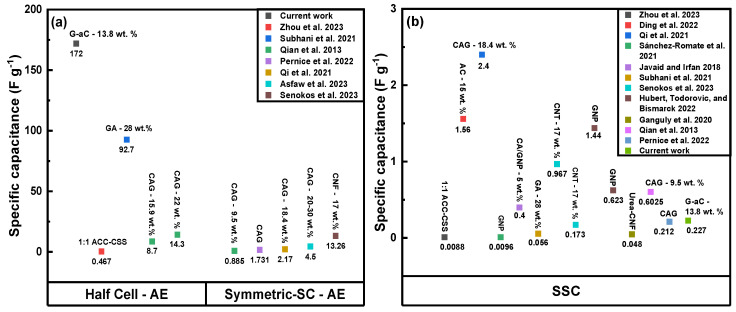
Comparison of the specific capacitance of the current work with the others; (**a**) functionalized CF-based electrodes (in aqueous electrolytes [AE]) [3,14,23,24,28,69,72] and (**b**) respective SSC devices (with SPE) [3,21,23,24,25,27,28,63,68,69,72].

**Figure 12:**
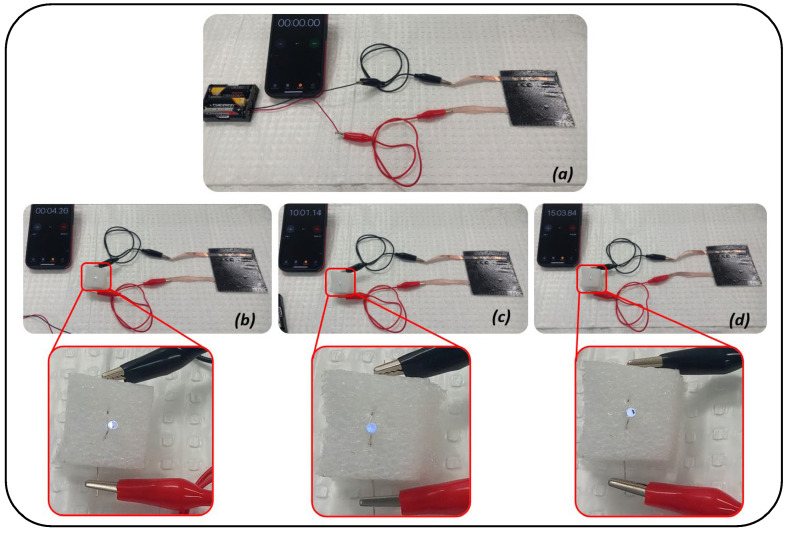
Charged SSSC powering an LED; (**a**) no light before connection, (**b**) bright light at the beginning of the connection, (**c**) moderately illuminating LED after 10 min of connection, (**d**) dim lighting of LED after 15 min of connection.

Although the electrochemical performance of the SSSC is lower than that of the G-aC/CF fiber, the supercapacitor demonstrates a superior capacitance value compared to previously reported studies on modified CFs [3,23,25,28,68,69]. Furthermore, it achieved a higher energy density than the results noted in [3,23,24,25,27,32,63,68,69,70], as well as a higher power density, comparable to that of [3,24,25,28,32,68,70], as displayed in Figure 11a,b and listed in Table 2. The decline in capacitance of the supercapacitor device compared to that of coated fibers is attributed to the low conductivity of the SPE between electrodes and resistance between electrodes, electrolyte, and separator in comparison to the highly conductive 6M KOH electrolyte used to test the coated fiber tows [28,71]. Additionally, our proof-of-concept SSSC device performance was assessed by charging it for 2 h by connecting it to a 4.5 V power supply. Then, the charged SSSC device was used to illuminate (see Figure 12) an LED for 15 min, as shown in the Supplementary Video S1. Cycling studies were performed over 10,000 cycles at 100 mV s^−1^, as shown in Figure 10d. A 10 % increment in performance is observed between 4000 and 5000 cycles, and by the end of 10,000 cycles, an excellent capacity retention of 97.3 % is observed for the SSSC.

**Figure 13:**
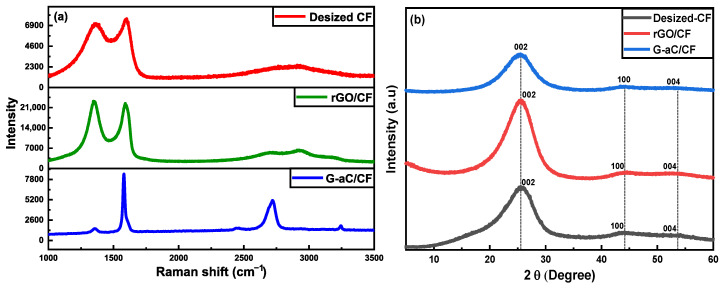
(**a**) Raman and (**b**) XRD Spectra of desized CF, rGO/CF, and G-aC/CF.

Raman spectroscopy was conducted on desized CF, rGO/CF, and G-aC/CF to characterize the irregularities and arrangement of the carbon-based materials. It can be seen from the spectra in Figure 13a that desized CF has two distinct peaks across the region at 1364 cm^−1^ and 1600 cm^−1^, denoting D and G bands, respectively. Similarly, rGO/CF have two broad peaks corresponding to D and G bands across the region at 1352 cm^−1^ and 1589 cm^−1^, respectively. Here, the D peak represents the amorphous form corresponding to sp^2^-hybridized out-of-plane vibrations due to structural defects of carbon, and G bands denote the graphitic form corresponding to the sp^2^-network in-plane vibrations of carbon with defects or disorders, respectively [73,74]. Seemingly, the intensities of the D and G bands of the rGO/CF are higher than that of desized CF, denoting the presence of the defective carbon network introduced by the surface coating of the GO through the hydrothermal process [75]. Moreover, wider bands represent the surface defects introduced due to the oxygen functional groups of GO and the higher intensity of the D band, denoting the disorderliness at the edges of the carbon network [73]. It is apparent that the spectra of G-aC/CF, with the D region at 1356 cm^−1^ and G-band at 1578 cm^−1^, possess lower intensity peaks denoting defects that are decreased due to the reduction of graphene oxide. Second-order characteristic peaks of the G-aC/CF_2_ are positioned around the region at 2462 cm^−1^, 2720 cm^−1^, and 3244 cm^−1^ affiliated with the G*, 2D(G′), and G″(2D′) bands, respectively. They are associated with the crystalline form of graphite, based on the research findings provided by Kaniyoor et al. [76], where the single low-intensity peak of the 2D band with two peak profiles with a small shoulder on its profile reveals the stacked graphene layers of up to five or six. In addition, the intensity of the 2D band is lower than that of the G band, confirming that single-layer graphene is not formed on the surface of CFs. If the Tuinstra–Koenig relation of the intensity ratio (I_D_/I_G_) for D to G bands is assessed, then the rGO/CF has a higher value of 1.008 than the value of 0.85 for the desized CF, revealing that defects are still present for the rGO layers, even after GO is reduced through the hydrothermal process. Similar to the work of Chen et al., the carbon lattice is converted into the graphitic structure by expelling hydrogen and oxygen atoms from sp^3^ carbons by forming more sp^2^ domains, which increases the ratio of I_D_/I_G_. This indicates that the reduction of GO does not completely clear away the defects [77].

Additionally, XRD analysis was conducted on fabric samples of desized CFs, rGO/CF, and G-aC/CF. The XRD spectra for desized CFs revealed a characteristic intense peak at approximately 2θ = 25.50°, accompanied by two weaker peaks at 2θ = 44.0° and 2θ = 54.0°. These peaks correspond to the 002, 100, and 004 crystal planes of graphite [78,79,80], as illustrated in Figure 13b. Similarly, the rGO/CF sample exhibited peaks at 2θ = 25.60°, 2θ = 44.5°, and 2θ = 54.0°. The G-aC/CF sample also showed peaks at 2θ = 25.50°, 2θ = 44.25°, and 2θ = 53.25°, which relate to the crystal planes of 002, 100, and 004, respectively [81,82]. The interlayer distance (d) between carbon and graphene sheets of the samples is determined, by using Bragg’s law (nλ = 2dsi nθ), to be 0.34 nm, which agrees with the typical value of d for carbonaceous materials, where n = 1 (order of reflection), λ = 0.15418 nm (X-ray diffraction), and θ is the angle of diffraction obtained from spectra in degrees [83,84]. The typical interlayer distance among adjacent graphene sheets in graphene oxide is around 0.9 nm, with a diffraction peak observed around 2θ = 10°, as reported elsewhere [85,86]. After reduction via hydrothermal and high-temperature treatment, the d spacing was observed to be 0.34 nm, with a shift in position of the diffraction peak to 2θ = 25.6°, indicating that the reduction of GO facilitated the removal of oxygen functional groups by reducing the interlayer distance, leading to restacking of rGO sheets [81]. This conclusion is further corroborated by Raman spectroscopy, which reveals a decrease in the intensity of the D band following heat treatment [87].

**Figure 14:**
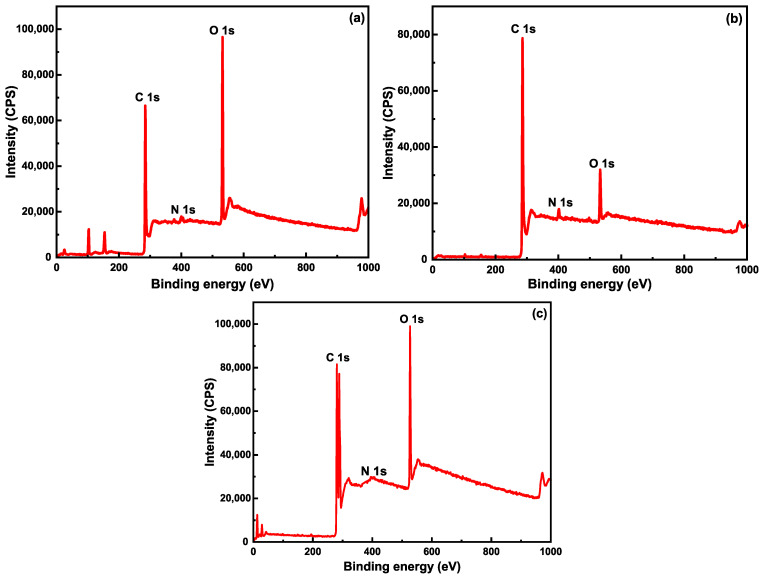
XPS wide scan spectra of samples; (**a**) desized CF, (**b**) rGO/CF, and (**c**) G-aC/CF.

Further, samples of desized CF, rGO/CF, and G-aC/CF were studied using X-ray photoelectron spectroscopy (XPS) to analyze the composition of the surface elements and existing functional groups. Wide scan spectra of samples are shown in Figure 14a–c, with major peaks of carbon (C1s) and oxygen (O1s) being detected with relatively less intense peaks for nitrogen (N1s). Furthermore, additional peaks related to silicon (see Figure S4) were observed, as the samples were loaded onto silicon wafers during the analysis (a comprehensive explanation can be found in the Supporting Information). Atomic concentrations of carbon, oxygen, and nitrogen in samples of desized CF, rGO/CF, and G-aC/CF are presented in Table 3. Comparing the atomic concentrations of elements in wide scan spectra of desized CF and rGO/CF reveals that the carbon content had increased from 71.5 at% to 88.83 at% with a decrease in oxygen content from 27.12 at% to 8.23 at%, respectively, due to the accumulation of rGO sheets onto the surface of CFs. Moreover, the oxygen content of sample G-aC/CF (14.09 at%) is comparatively higher than that of rGO/CF (8.23 at%), revealing that activating the graphene with KOH has introduced more oxygen groups on the graphene layers. The nitrogen content increased after coating with GO, followed by a reduction, from 1.73 at% to 2.88 at%. The nitrogen-containing groups were removed by high-temperature activation [88] and reduced to the minimum amount of 0.14 % when forming G-aC/CF, indicating that CFs are well-coated with graphene and activated carbon [89].

**Figure 15:**
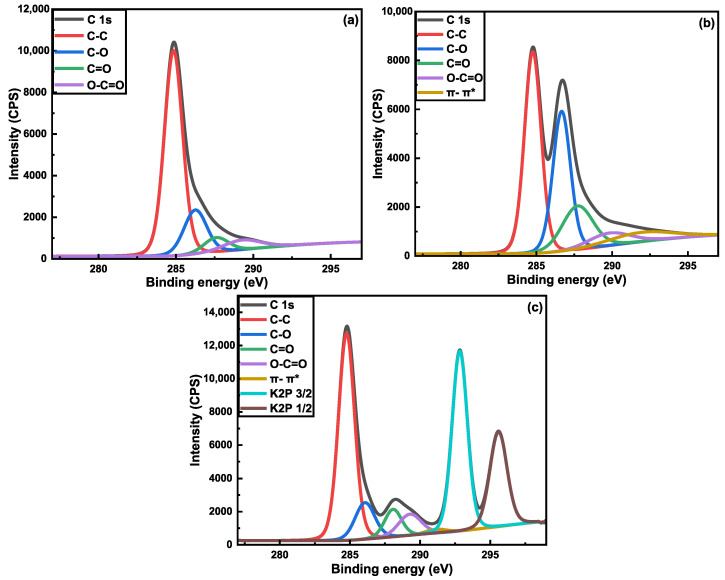
XPS high-resolution spectra of C1s with deconvoluted peaks for samples of (**a**) desized CF, (**b**) rGO/CF, and (**c**) G-aC/CF.

High-resolution XPS scans of the C1s region for samples of desized CF, rGO/CF, and G-aC/CF are shown in Figure 15a–c. The deconvoluted C1s high-resolution spectra of desized CF in Figure 15a show various peaks for the functional groups of C-C (69.28 at%), C-O (16.3 at%), C=O (6.62 at%), and O-C=O (7.8 at%), with corresponding binding energies (B.E) of 284.85, 286.26, 287.54, and 288.91 eV [90], respectively. The increased oxidized carbon composition of nearly 30 at% can be attributed to the treatment of the CFs with nitric acid [91]. After coating the CFs with GO, followed by hydrothermal reduction, the deconvoluted C1s peaks for rGO/CF, as shown in Figure 15b, are shown to have functional groups of C-C (40.41 at%), C-O (29.82 at%), C=O (16.22 at%), O-C=O (6.41 at%), and π-π* satellite bonds, with corresponding binding energies of 284.77, 286.67, 287.72, 289.81, and 291.89 eV, respectively [73,92]. Similarly, the deconvoluted C1s peaks for G-aC/CF, as shown in Figure 15c, are shown to have functional groups of C-C (34.99 at%), C-O (7.61 at%), C=O (4.97 at%), O-C=O (5.07 at%), π-π* satellite bonds, K2P 3/2 (29.11 at%), and K2P 1/2 (17.24 at%), with corresponding binding energies of 284.77, 286.08, 288.08, 289.28, 291.4, 292.83, and 295.59 eV, respectively. It can be observed that after the reduction of GO on the CF, oxygen-containing groups have increased, indicating that the GO has not been fully reduced, and that the rGO does not possess the structure of pristine graphene. After high-temperature treatment of the slurry-coated CFs, there is an effective reduction of GO by eliminating oxygen groups, as the numbers suggest (see Table 3). Moreover, two peaks with higher B.E, corresponding to K2P 3/2 (29.11 at%) and K2P 1/2 (17.24 at%), are introduced due to the existence of impurities in the form of potassium from using KOH to activate the graphene while preparing the slurry [93,94]. Table 4 shows the corresponding B.E and atomic concentrations of various functional groups of the samples analyzed.

## 4. Conclusions

In summary, desized CFs were functionalized with graphene and aC. After performing a dual coating procedure via hydrothermal and brush coating, high-temperature activation produced G-aC/CF electrodes. Surface etching created a coarse surface with grooves and increased oxygen functional groups, leading to improved bonding with the coating material. The formation of a well-connected network of rGO sheets, including the creation of a porous structure induced by aC, not only increased the surface area, but also facilitated better electrical conductivity and enhanced ionic movement for the electrolytic ions. The aC acts as a spacer among graphene sheets that prevents the agglomeration of graphene sheets and increases the surface area. The fabrication of an SSSC device with G-aC/CF electrodes exhibited a significant improvement in the surface area of nearly 210 times greater than that of uncoated fibers. This is accompanied by the SEM images, revealing that the CFs are adequately coated with the formation of a porous structure. Both the coated fiber electrode and the as-prepared SSSC device exhibit excellent performance in terms of enhanced capacitance and a wide operational window, with values of 172 F g^−1^ across 1.5 V, and 227 mF g^−1^ across 6 V, respectively. Further, the as-prepared SSSC device exhibits remarkable cycling stability, retaining 97.3% of its capacity over 10,000 cycles. The coated fiber electrode also demonstrated energy and power densities of 44.4 Wh kg^−1^ and 150 W kg^−1^, respectively, at a current density of 0.2 A g^−1^. As a proof-of-concept for the SSSC device, it was demonstrated that it could power an LED for 15 min. The performance of the SSSC is constrained by the ion conductivity of the currently available solid polymer electrolytes. Nevertheless, the significant improvement observed in the performance of the SSSC device made from G-aC/CF electrodes highlights the effectiveness of the method employed in this study. This approach presents a viable process for producing SSCs suitable for application in future structural components in the aerospace and automotive industries, potentially contributing to weight savings while enhancing payload and range capabilities.

## Figures and Tables

**Table 1:** Comparison of the performance of desized CFs with coated CFs and coating material relative to BET data.

Sample Material	Loading of Active Material on CF (wt.%)	BET Surface Area (m^2^ g^−1^)	Pore Volume (cm^3^ g^−1^)	Pore Width (nm)	Specific Capacitance (F g^−1^)
Desized—CF	-	0.16	0.0015	8.5	9.38
rGO/CF	3.2	2.36	0.0020	2.46	22.54
G-aC/CF_2_	13.8	34.21	0.0190	2.25	172
G-aC powder	-	64.27	0.0650	4.15 ± 0.25	-

**Table 2:** Comparison of the current work with the capacitance of other functionalized CF-based electrodes and respective SSC devices.

Electrode	Material Loading	Device Type	Electrolyte	Capacitance (C)	Energy Density (E)	Power Density (P)	References
G-aC/CF	13.8 wt.%	Three-electrode cell	6 M KCl	172 F g^−1^	44.36 Wh kg^−1^	150 W kg^−1^	Current work
		SSC	PEGDGE (83 wt.%), EMIMTFSI (10 wt.%), TETA (7 wt.%), 2 g of LiFSI	0.227 F g^−1^	0.9897 Wh kg^−1^	3.5 W kg^−1^	
CF Fabric—CAG	22 wt.%	Three-electrode cell	3 M KCl	14.3 ± 0.2 F g^−1^	-	-	[24]
	15.9 wt.%	Three-electrode cell	3 M KCl	8.7 ± 0.3 F g^−1^	-	-	
	9.5 wt.%	SSC	EMITFSI	0.885 F g^−1^	1230 µWh kg^−1^	420 µW kg^−1^	
		SSC	82.6 wt.% PEGDGE,10 wt.% EMITFSI, 7.4 wt.% TETA	0.602 F g^−1^	840 µWh kg^−1^	32.8 µW kg^−1^	
GNP-WCF	-	SSC	33.75 wt.% of LY, 33.75 wt.% of a PEGDGE, 30 wt.% of EMITSFI + TiO_2_, DDS hardener	0.0096 F g^−1^	0.00286 Wh kg^−1^	1.139 W kg^−1^	[25]
CF-CA/GNP	5 wt.%	SSC	DGEBA (43.9 wt.%), 1 M lithium perchlorate in propylene carbonate (50 wt.%), triethylene tetramine (6.1 wt.%)	0.4 F g^−1^	0.8 Wh kg^−1^	108 W kg^−1^	[27]
CF fabric - GA	28 wt.%	Three-electrode cell	6 M KCl	92.7 F g^−1^	1 Wh kg^−1^	677 W kg^−1^	[28]
		SSC	PEDGE (75 wt.%), TETA (11.4 wt.%) and EMITFSI (13.6 wt.%)	0.0056 F g^−1^	-	0.0225 W kg^−1^	
AC/WCF	15 wt.%	SSC	DGEBA:AG80 (7:3), EMIM-TFSI, D400 polyether amine	1.56 F g^−1^	1.53 Wh kg^−1^	42.66 W kg^−1^	[21]
CAG-Spread tow CF	20–30 wt.%	Symmetrical SC	EMI-TFSI	4.5 F g^−1^	2.6 Wh kg^−1^	440 W kg^−1^	[14]
1:1 ACC-CSS	-	SSC	PVA-KOH gel electrolyte	0.0088 F g^−1^	0.0099 Wh kg^−1^	0.4455 W kg^−1^	[3]
		Three-electrode cell	1 M KOH	0.467 F g^−1^	-	-	
MnOOH-NWs@WCF	-	SSC	EMIMTFSI (69 wt.%), LiTFSI (30 wt.%), PC (1 wt.%), Epoxy resins (E-51 and PEGDGE), D-230	0.0771 F cm^2^	0.0812 Wh kg^−1^	0.04737 W kg^−1^	[70]
PANI-CF	0.05 mg cm^−2^	SSC	DGEBA (43%), LiClO_4_ (50%), PC, TETA (7%)	0.02005 F cm^2^	0.0446 Wh kg^−1^	1.59 W kg^−1^	[32]
PANI-ACF	0.05 mg cm^−2^	SSC		0.02224 F cm^2^	0.0494 Wh kg^−1^	21.63 W kg^−1^	
CF-Urea-GNF	-	SSC	PEGDGE/EMIMTFSI	0.048 F g^−1^	7 × 10^−5^ Wh kg^−1^	0.788 W kg^−1^	[68]
CAG-CF	18.4 wt.%	SSC	BADGE, IPDA, EMIM-TFSI	2.4 F g^−1^	2.15 Wh kg^−1^	98.8 W kg^−1^	[72]
		Symmetrical SC	EMIM-TFSI	2.17 F g^−1^	1.55 Wh kg^−1^	61.7 W kg^−1^	
CNT-CF	17 wt.%	SSC	PEGDGE, EMIMTFSI, TETA	0.173 F g^−1^	0.055 Wh kg^−1^	17 W kg^−1^	[23]
			PEGDGE, EMIMTFSI + additional 6 wt.% IL, TETA	0.967 F g^−1^	0.3 Wh kg^−1^	84.5 W kg^−1^	
		Symmetrical SC	EMIMTFSI	13.26 F g^−1^	3.8 Wh kg^−1^	57,789 W kg^−1^	
CAG-CF	-	SSC	DGEBA (47.2 wt.%), IPDA 11.8 wt.%, EMIM-TFSI (41 wt.%)	0.212 F g^−1^	0.093 Wh kg^−1^	5.2 W kg^−1^	[69]
		Symmetrical SC	EMIMTFSI	1.731 F g^−1^	1.405 Wh kg^−1^	301 W kg^−1^	
CF-GNP	-	SSC	PEGDGE (82.6 wt.%), TETA (7.4 wt.%) and EMIBF_4_ (10 wt.%)	0.623 F g^−1^	0.0169 Wh kg^−1^	5.2 W kg^−1^	[63]
				1.44 F g^−1^	-	-	

**Table 3:** XPS wide scan analysis with an atomic concentration of C1s, O1s, and N1s in desized CF, rGO/CF, and G-aC/CF.

Sample	Element	Atomic Concentrations (at%)
Desized CF	C 1s	71.5
	O 1s	27.12
	N 1s	1.73
rGO/CF	C 1s	88.83
	O 1s	8.23
	N 1s	2.88
G-aC/CF	C 1s	85.77
	O 1s	14.09
	N 1s	0.14

**Table 4:** XPS high-resolution spectra of C1s with surface functional groups with various parameters for samples of desized CF, rGO/CF, and G-aC/CF.

Samples	Surface Functional Group	Binding Energy	Atomic Concentrations
Desized CF	C-C	284.85	69.28
	C-O	286.26	16.3
	C=O	287.54	6.62
	O-C=O	288.91	7.8
rGO/CF	C-C	284.77	40.41
	C-O	286.67	29.82
	C=O	287.72	16.22
	O-C=O	289.81	6.41
	π-π*	291.89	7.41
G-aC/CF	C-C	284.77	34.99
	C-O	286.08	7.61
	C=O	288.08	4.97
	O-C=O	289.28	5.07
	π-π*	291.4	1.01
	K2P 3/2	292.83	29.11
	K2P 1/2	295.59	17.24

## Supplementary Materials

The following supporting information can be downloaded at: https://www.mdpi.com/article/10.3390/nano15171325/s1, A supporting information document and a video showing the working of the proof-of-concept SSC is provided along with this manuscript. Refs. [95,96] are cited in the Supplementary Materials.

## Abbreviations

The following abbreviations are used in this manuscript:

G	Graphene
GO	Graphene oxide
rGO	Reduced graphene oxide
LED	Light-emitting diode
SSC	Structural supercapacitor
SC	Supercapacitor
ESD	Energy storage device
ESC	Energy storage composites
EDLC	Electric double layer capacitor
CF	Carbon fiber
GA	Graphene aerogel
CFRP	Carbon fiber-reinforced polymer
CNT	Carbon nanotubes
PAN	Polyacrylonitrile
PPy	Polypyrrole
CAG	Carbon Aerogel
GNP	Graphene nanoplatelets
aC	Activated carbon
HCL	Hydrochloric acid
KOH	Potassium hydroxide
EG	Ethylene glycol
PEGDGE	Polyethylene glycol diglycidyl ether
TETA	Triethylenetetramine
EMIMTFSI	1-Ethyl-3-methylimidazolium bis(trifluoromethylsulfonyl)imide
LiFSI	lithium bis(fluorosulfonyl)imide
VRTM	vacuum-assisted resin transfer molding
SEM	Scanning Electron Microscope
CV	Cyclic voltammetry
BET	Brunauer–Emmett–Teller
XPS	X-ray photoelectron spectroscopy
