# Supplementary material for: Nanomaterial Functionalized Carbon Fiber-Reinforced Composites with Energy Storage Capabilities
Source: Nanomaterials (Basel). 2025 Aug 28;15(17):1325. doi: 10.3390/nano15171325 (PMC12429961; doi:10.3390/nano15171325)
Supplement: Supplementary file 1 [file nanomaterials-15-01325-s001.zip › nanomaterials-3735613-supplementary.pdf]

Supporting information

Nanomaterial functionalized carbon fiber-reinforced composites with energy storage capabilities

Venkatesh Gangipamula[1,2], Karamat Subhani[1], Peter J. Mahon[3], Nisa Salim[1,*]

[1] School of Engineering, Swinburne University of Technology, Melbourne, VIC 3122, Australia
[2] Aerostructures Innovation Research (AIR) Hub, Swinburne University of Technology, Melbourne, VIC 3122, Australia
[3] School of Science, Computing and Emerging Technologies, Swinburne University of Technology, Melbourne, VIC 3122, Australia
* Correspondence: nsalim@swin.edu.au (N.V. Salim)

S1. Results and discussion

SEM imaging of nano-form-cellulose reveals its structure in the form of material pieces with varying sizes (see Figure S1(a-c)). These pieces are brittle, which makes it possible to blend them with graphene. After activation, followed by high-temperature treatment, they formed into highly porous structures, as shown in Figure S1d.

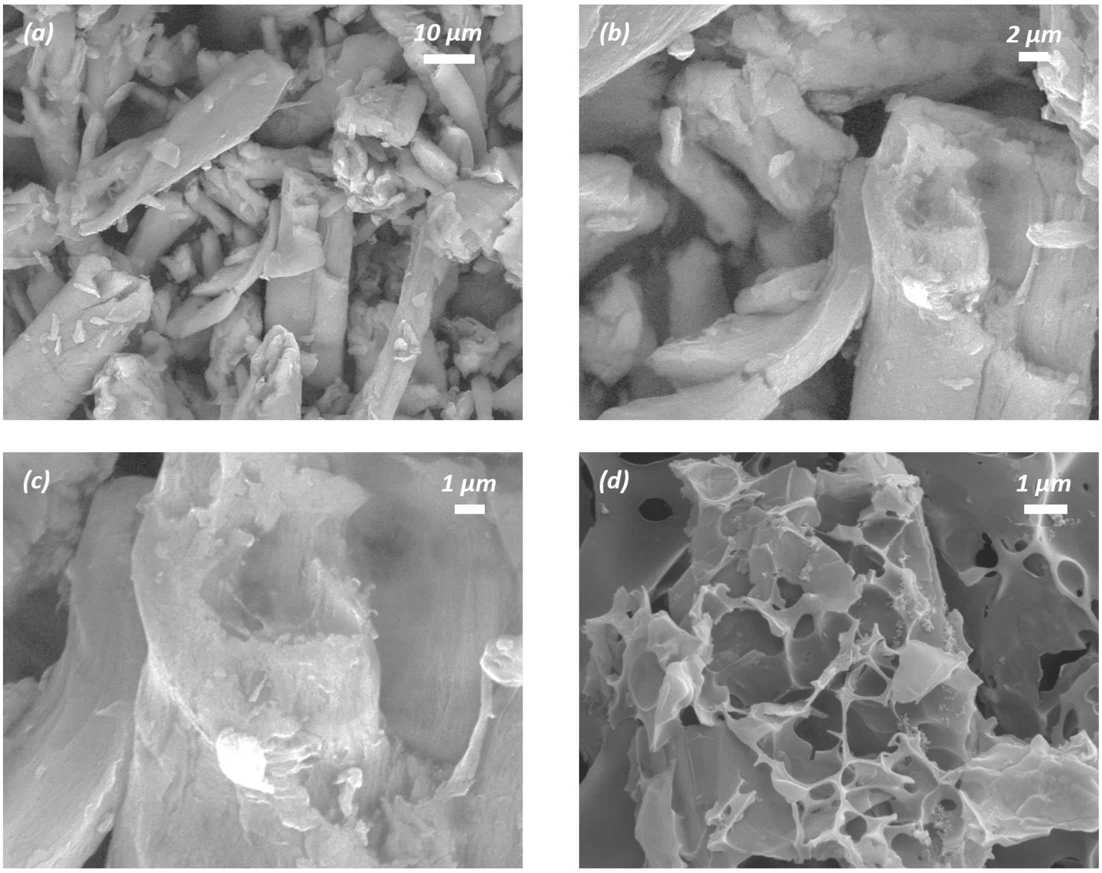

Figure S1. SEM imaging of (a, b, c) cellulose powder and (d) G-aC coating on CF.

The BET surface analysis of G-aC - powder exhibits a higher surface area of 64.27 m^2 g^{-1}, with almost double the value of G-aC/CF and a pore volume more than 3 times that of G-aC/CF

as shown in Figure S2. This is because powdered samples usually have loosely packed materials with wider spaces and the least bonding among them, making it feasible for more surface access for N_2 absorption and detection of larger pore distributions.

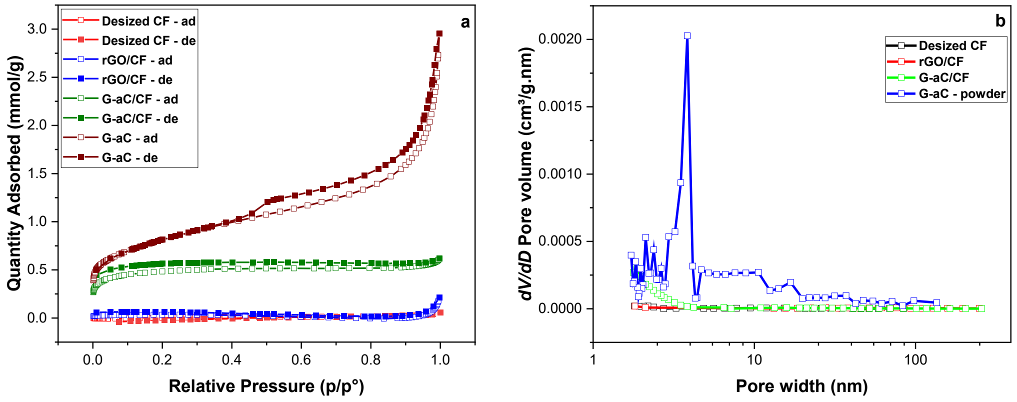

Figure S2. Nitrogen absorption (ad) and desorption (de) isotherms and (b) Pore size distribution of desized CF, rGO/CF, G-aC/CF and G-aC - powder

The specific capacitance results of desized CF, rGO/CF, G-aC/CF_1 and G-aC/CF_2 using cyclic voltammetry, illustrated by Figure S4(a-d), reveal that there is increasing performance after each coating step, the highest gravimetric capacitance at a scan rate of 1 mV s^{-1} for rGO coated CF is almost double that for desized CF, it was further increased by 3 times for a poor form of a G-aC coating. With the better composition of slurry, the performance was enhanced by 17 times that of desized CFs, as shown in Table S1.

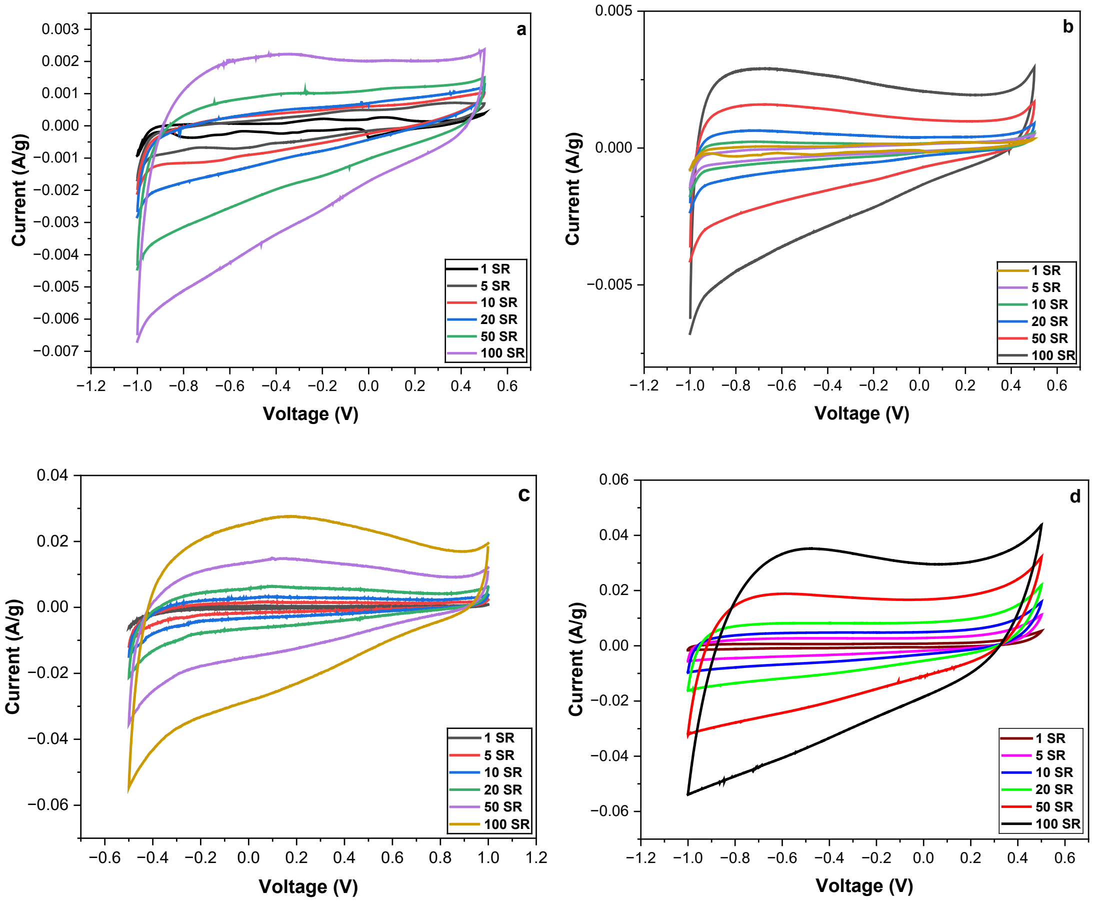

Figure. S3. Cyclic voltammograms of (a) desized CF, (b) rGO/CF, (c) G-aC/CF_1 and (d) G-aC/CF_2 fiber tow at different scan rates).

Table S1. Comparison of the Specific capacitance of desized CFs with coated CFs in 6 M KCl electrolyte at different scan rates.

	Specific capacitance (F/g)			
**Scan rate ($mV.s^{-1}$)**	**Desized CF**	**rGO/CF**	**G-aC/CF_1**	**G-aC/CF_2**
100	1.48	4.47	17.74	59.52
50	1.65	4.78	21.65	72.02
20	2.12	4.96	23.32	90.37
10	3.05	4.95	23.74	106.03
5	4.44	5.57	24.25	123.02
1	9.38	22.54	29.93	172.07

The XPS wide scan spectrum displayed in Figure S4 illustrates the peaks associated with silicon, which serve as the substrate for the sample analysis. Multiple peaks corresponding to silicon are evident, although they exhibit negligible intensities. Characteristic peaks related to the Si 2p orbitals, exhibiting a splitting of $2p_{½}$ - $2p_{3/2}$, are observed at a binding energy of 102 eV, indicating the presence of pure silicon. Furthermore, peaks corresponding to Si2s and Si3s appear at binding energies of 151 eV and 12.8 eV, respectively [1]. Minor peaks related to O2s and OKLL are also detected, attributed to lattice oxygen in silicon dioxide, though no significant atomic concentrations are identified [2].

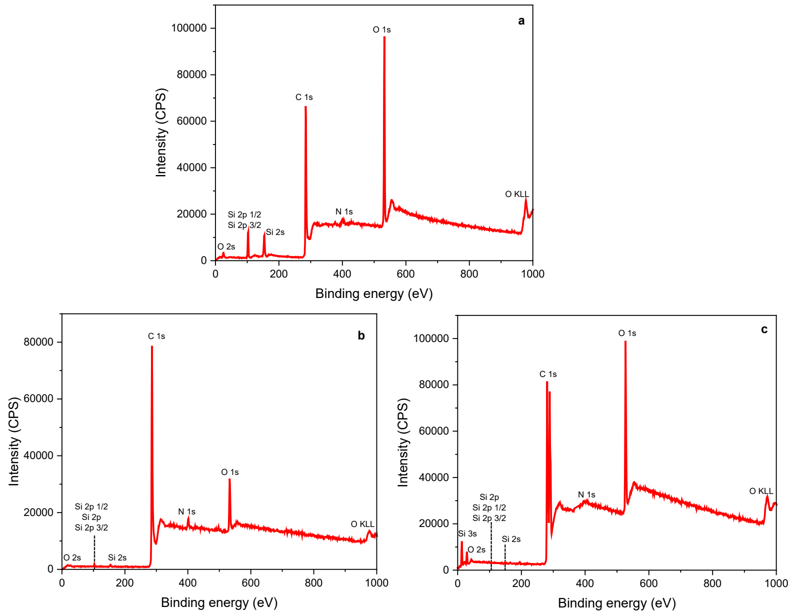

Figure S4. XPS Wide scan spectra of samples with all elements (a) desized CF, (b) rGO/CF, and (c) G-aC/CF.

References

1. Barr, T.L., *An XPS study of Si as it occurs in adsorbents, catalysts, and thin films.* Applications of Surface Science, 1983. **15**(1-4): p. 1-35.
2. Jensen, D.S., et al., *Silicon (100)/sio2 by xps.* Surface Science Spectra, 2013. **20**(1): p. 36-42.
